# The importance of registration of sexual orientation and recognition of indicator conditions for an adequate HIV risk-assessment

**DOI:** 10.1186/s12879-017-2279-y

**Published:** 2017-02-28

**Authors:** Ivo K. Joore, Suzanne E. Geerlings, Kees Brinkman, Jan E. A. M. van Bergen, Jan M. Prins

**Affiliations:** 1 Department of General Practice, Division Clinical Methods and Public Health, Academic Medical Center, Meibergdreef 9, 1100 DE Amsterdam, The Netherlands; 2 Department of Internal Medicine, Division of Infectious Diseases, Academic Medical Center, Amsterdam, The Netherlands; 3 Department of Internal Medicine, OLVG, Amsterdam, The Netherlands; 4 STI AIDS Netherlands (SOA AIDS Nederland), Amsterdam, The Netherlands

## Abstract

**Background:**

HIV testing among risk groups and guided by HIV indicator conditions (IC) is widely recommended by European guidelines. In this study we investigated how these strategies are used by general practitioners (GP) and in other healthcare settings. The objectives of our study were to describe: 1) the proportion of consultations in primary care and other healthcare settings in the five years prior to diagnosis; 2) patient and GP perspectives on the primary healthcare providers’ awareness and registration of sexual orientation and ethnicity in the electronic medical record (EMR); and 3) the proportion of HIV-infected patients who had been diagnosed with an IC prior to HIV diagnosis.

**Methods:**

A survey study (2014–2016) was conducted among newly diagnosed HIV-infected patients presenting at two HIV outpatient clinics in Amsterdam. We collected information on the number of consultations and extent of HIV testing in healthcare settings in the 5 years prior to HIV diagnosis; on patient and GP perspectives on the primary healthcare providers’ awareness of sexual orientation and ethnicity; and on preselected ICs and symptoms of acute HIV infection prior to diagnosis. GPs were also approached for further information.

**Results:**

In the 5 years prior to HIV diagnosis, 82.9% of the 111 patients had one or more consultations with their GP, but only 34.8% had one or more HIV tests performed in general practice during this period. In more than 50% of cases the patients took the initiative for the positive HIV test. GPs stated that they were aware of the sexual orientation of 59.6% of their patients who were men who have sex with men (MSM); however, sexual orientation was only documented in the EMR in 34.0% of these cases. GPs also reported that they were aware that a patient was from an HIV endemic country in more than half of the cases. GPs diagnosed 48.3% of all ICs and 39.5% of this group was offered an HIV test at that time.

**Conclusions:**

Documentation of sexual orientation and ethnicity, and IC-guided testing by GPs could be the starting point for more proactive provider-initiated HIV testing.

## Background

The number of people newly diagnosed with Human Immunodeficiency Virus (HIV) in the Netherlands is still approximately 1000 per year, the majority of whom (69%) are men who have sex with men (MSM) [1]. In 2014, 44% of those newly diagnosed with HIV in the Netherlands had presented late for care (CD4 count < 350 cells/mm^3^ or with an AIDS-defining event) [1]. Individuals from HIV endemic countries had a higher likelihood of presenting late for care [1]. This percentage was higher among females (53%) and heterosexual males (66%) from HIV endemic countries than in the MSM population (36%) [2]. Initiating treatment after HIV diagnosis, regardless of CD4 count, improves the prognosis of the person being treated and reduces transmission [3–5]. These findings show the importance of early detection and linkage to care.

Provider-initiated HIV testing among risk groups is widely recommended by guidelines on sexually transmitted infections (STIs)/HIV [6, 7]. A Dutch study showed that 75% of the HIV-infected patients presenting late for care had one or more risk factors for HIV recorded in the national STI guidelines [8]. Therefore, gathering information about patient sexual orientation or ethnicity is important for a better HIV risk assessment [6]. General practitioners (GPs) do not always collect this information from their patients [9].

In order to reduce the number of undiagnosed cases of HIV, as well as people presenting late for care, we need to explore other provider-initiated HIV testing strategies. HIV testing guided by indicator conditions (ICs) is a new provider-initiated HIV testing strategy [7, 10]. The European Centre for Disease Prevention and Control (ECDC) recommends the routine offer of an HIV test in patients with an IC, defined as: *“1) conditions which are AIDS-defining among people living with HIV; 2) conditions associated with an undiagnosed HIV prevalence of > 0.1%; and 3) conditions where not identifying the presence of HIV infection may have significant adverse implications for the individual’s clinical management despite the estimated prevalence of HIV most likely being lower than 0.1% – for example, when aggressive immune-suppressive therapy is considered (cancer or organ transplantation)”* [10]. A previous study conducted in Southeast Amsterdam showed that prior to diagnosis, 58.8% of all HIV cases presented in primary care with one or more IC, compared to 7.4% of controls, revealing possible opportunities for more provider-initiated HIV testing [11]. One important IC is mononucleosis-like illness, which could indicate an acute HIV infection, as 50 to 90% of people with the latter develop a mononucleosis-like illness, which is often mistaken for flu or acute Epstein Barr virus infection [12, 13].

HIV testing among risk groups and HIV IC-guided testing are widely recommended by international and national guidelines [6, 7, 10]. In this study of newly diagnosed HIV-infected patients we collected more information about how these strategies are used by GPs. The objectives of our study were to describe: 1) the proportion of consultations in primary care and other healthcare settings in the 5 years prior to diagnosis; 2) patient and GP perspectives on the primary healthcare providers’ awareness and registration of sexual orientation and ethnicity in the electronic medical record (EMR); and 3) the proportion of HIV-infected patients with an IC diagnosed by their GP prior to HIV diagnosis.

## Methods

### Data source

A survey study was conducted between January 2014 and April 2016 at the HIV outpatient clinics of two hospitals located in Amsterdam, the Netherlands. Patients newly diagnosed with HIV were eligible if they were 18 years or older and presenting for the first time at these clinics.

We collected information by using paper questionnaires which addressed demographic characteristics (gender, age), history of HIV testing, and number of consultations in healthcare settings in the 5 years prior to HIV diagnosis. The HIV-infected patients were also asked if their GP was aware of their sexual orientation. Furthermore, we collected information about ICs diagnosed in a healthcare setting in the 5 years prior to diagnosis [10].

The ECDC list of ICs was too long to be incorporated in our questionnaire, and we therefore preselected a small number of ICs (STIs, seborrheic dermatitis, oral candidiasis, herpes zoster, cervix dysplasia and pneumonia) that are observed in general practice. We also included tuberculosis and HIV-related cancers.

Patients with a recent HIV infection (a negative or undeterminate HIV test result less than 6 months prior to diagnosis) were asked whether they had experienced a mononucleosis-like illness in the 6 months prior to diagnosis; whether this illness was different to other flu-like illnesses in the past; and whether they had contacted their GP about this illness.

The HIV-infected patients were recruited by HIV nurse practitioners experienced in counselling. GPs were approached by phone for more information to determine their awareness and registration of the patient’s sexual orientation and, if applicable, whether they were aware that the patient originated from an HIV endemic country. GPs were approached by one of the researchers (IJ) involved in the study.

Additional information about the newly diagnosed HIV-infected patients included in our study was obtained from the ATHENA national observational HIV cohort in the Netherlands, which monitors all people registered as HIV positive in the 30 HIV treatment centres in the Netherlands, including four paediatric centres [1]. Linkage to the ATHENA database was done using an anonymized patient identifier. Additional data included sociodemographic data (country of origin), degree of urbanization, socioeconomic status (SES), clinical data and CD4 cell count at the time of HIV diagnosis.

Level of urbanization and socioeconomic status were defined as previously described [1, 2]. CD4 count at the time of diagnosis was defined as the first CD4 count within 3 months after diagnosis.

The medical ethics committees of both hospitals declared that, according to the Medical Research Involving Human Subjects Act (WMO), formal approval for this research project by a medical ethics committee was not required. The research was performed according to strict guidelines for the privacy protection of patients and GPs. All participants received information about the study and gave their written consent.

### Data analysis

Descriptive statistical methods were performed using SPSS (SPSS version 19.0 software, IBM, USA).

## Results

### Sociodemographic characteristics at HIV diagnosis

In total, 111 people were included in the study. Baseline characteristics are presented in Table 1. The majority of newly diagnosed HIV-infected patients (60.4%) were MSM, while 19.4% (13/67) of this group (MSM), as well as 66.7% (12/18) of females and 38.5% (10/26) of heterosexual males, originated from HIV endemic countries. Most were from moderate to highly urbanized areas and 58.5% had a higher than average SES status.

**Table 1:** Sociodemographic characteristics of newly diagnosed HIV-infected patients

Total	Number	Percent
	111	
Age at diagnosis, years		
18–29	24	21.6
30–49	56	50.5
≥ 50	31	27.9
Gender and sexual orientation		
MSM	67	60.4
Female	18	16.2
Heterosexual male	26	23.4
Originates from an HIV endemic country^a^		
Yes	35	31.5
No	76	68.5
Level of urbanization		
Highly urbanized	66	64.7
Urbanized	22	21.6
Moderately urbanized	7	6.9
Less urbanized	5	4.9
Rural	2	2.0
Missing	9	
Socioeconomic status		
Very wealthy	4	4.0
Wealthy	28	27.8
Average	27	26.7
Less favoured	18	17.8
Deprived	24	23.8
Missing	10	

^a^Patients born in the Caribbean, Sub-Saharan Africa, South or Southeast Asia, Latin/South America, North Africa, Middle East or Eastern Europe

### History of HIV testing

In the 5 years prior to HIV diagnosis, 21/109 patients (19.3%) had not been tested for HIV (Table 2). In terms of sociodemographics this can be broken into: 10.6% (7/66) of the MSM group, 27.8% (5/18) of females, 36.0% (9/25) of heterosexual males, and 25.7% (9/35) of patients originating from HIV endemic countries.

**Table 2:** History of HIV testing

Total	Number	Percent
	111	
History of HIV testing		
Never tested	21	19.3
Tested negative prior HIV diagnosis	86	78.9
Not sure	2	1.8
Missing	2	
CD4 count at entry into care (cells/mm3)		
≤ 350	40	37.4
350–500	21	19.6
≥ 500	46	43.0
Missing	4	
Initiative for HIV positive test		
Patient	62	56.4
Healthcare provider	48	43.6
Missing	1	
Diagnosed at (setting)		
General Practice	31	27.9
STI outpatient clinic	38	34.2
Hospital	29	26.1
Other	13	11.7
Reason for testing of the HIV positive test		
STI-related symptoms	56	51.9
Routine health check	23	21.3
Pregnancy	5	4.6
At risk, own risk perception	4	3.7
At risk: sex with known HIV positive sex partner	3	2.8
At risk: contacted by a sex partner who tested HIV positive	6	5.6
Anonymous notification of a sex partner of HIV (by STI outpatient clinic)		
Other	11	10.2
Missing	3	
Refusal of HIV test in the past		
Yes	1	0.9
No	110	99.1

At diagnosis, 40 HIV-infected patients (37.4%) had a CD4 cell count ≤ 350 cells/mm^3^: 29.2% (19/65) of the MSM group; 29.4% (5/17) of females; and 64.0% (16/25) of heterosexual males. Patients had taken the initiative for the HIV (positive) test in more than half of the cases (56.4%).

The STI outpatient clinic (34.2%), general practice (27.9%) and hospitals (26.1%) were all important settings for diagnosing the HIV infection. In more than half of the patients (51.9%), STI-related symptoms were the reason for HIV testing and 21.3% of the patients were diagnosed by a routine health check.

### Patient consultations in healthcare settings in the five years prior to HIV diagnosis

In the 5 years prior to HIV diagnosis, the majority (82.9%) of patients had one or more consultations with their GP, and two-thirds had three or more consultations (Table 3). More than half (55.9%) visited one or more medical specialists, 34.2% an emergency department and 50.5% the outpatient STI clinic. In the 5 years prior to diagnosis, 34.8% of those who had visited their GP had one or more HIV tests performed in the general practice, compared to 85.7% of those who visited an STI clinic.

**Table 3:** Consultations in healthcare settings in the 5 years prior to HIV diagnosis

Total	Number	Percent
	111	
General practitioner		
None	19	17.1
One consultation	11	9.9
Two consultations	9	8.1
Three or more consultations	72	64.9
One or more HIV tests performed prior to the HIV diagnosis	32	34.8
Medical specialist		
None	49	44.1
One medical specialist	34	30.6
Two medical specialist	16	14.4
Three or more medical specialists	12	10.8
One or more HIV tests performed prior to the HIV diagnosis	9	14.5
Emergency department		
None	73	65.8
One consultation	24	21.6
Two consultations	9	8.1
Three or more consultations	5	4.5
One or more HIV tests performed prior to the HIV diagnosis	4	10.5
STI outpatient clinic		
None	55	49.5
One consultation	16	14.4
Two consultations	8	7.2
Three or more consultations	32	28.9
One or more HIV tests performed prior to the HIV diagnosis	48	85.7

### GP knowledge and registration of sexual orientation in the Electronic Medical Record

For 13 MSM, no contact with the GP was possible and four people in this group refused permission to contact their GP. Of the remaining GPs, 59.6% (28/47) reported that they were aware of the sexual orientation of the patient (Table 4). In contrast, 70.8% (34/48) of the MSM group reported that the GP had knowledge of their sexual orientation.

**Table 4:** GP awareness and registration of sexual orientation and ethnicity in the electronic medical record

Total	Number	Percent
MSM	50	
GP awareness of sexual orientation		
Yes	28	59.6
No	19	40.4
Missing	3	
Patients’ perception that GP had knowledge about their sexual orientation		
Yes	34	70.8
No	14	29.2
Missing	2	
Sexual orientation was documented in the EMR of the GP		
Yes	16	34.0
No	31	66.0
Missing	3	
Person originated from an HIV endemic country	19	
GP aware that patient originated from an HIV endemic country		
Yes	9	56.3
No	7	43.8
Missing	3	

In 71.4% (20/28) of the MSM group of which the GPs were aware of their sexual orientation, no HIV test was performed in the general practice prior to diagnosis, compared to 78.9% (15/19) of the MSM group of which the GP was not aware of their sexual orientation. The sexual orientation was indeed documented in the EMR of the GP in 34.0% (16/47) of the MSM cases.

Information was also collected about GP awareness of patient origins in HIV endemic countries. For 14 patients from HIV endemic countries, no contact with the GP was possible and two patients refused permission to contact their GP. GPs reported that they were aware that the patient was from an HIV endemic country in 56.3% (9/16) of patients from this group.

### HIV indicator conditions and notification of GP of mononucleosis-like illness

More than half (56.8%) of the patients had one or more IC in the 5 years prior to HIV diagnosis: 35.1% (39/111) had one IC; 12.6% (14/111) had two ICs; and 9.0% (10/111) had three or more ICs. The most diagnosed ICs were STIs (68.0%), herpes zoster (11.7%) and seborrheic dermatitis (8.7%). GPs diagnosed 48.3% (43/89, excluding 14 missing variables) of the ICs; 13.5% (12/89) of ICs were diagnosed in hospitals; and 38.2% (34/89) in STI clinics. Of the patients diagnosed with an IC by the GP, 39.5% (17/43) were offered an HIV test.

In 26.1% (29/111) of the patients, the HIV infection was recent. Of these patients, 79.3% (23/29) were MSM or originated from an HIV endemic country. Of this group, 88.5% (23/26) reported that they had a mononucleosis-like illness in the 6 months prior to their diagnosis, with 73.9% (17/23) reporting that this illness was different to other flu-like illnesses in the past. The GP was contacted in 76.5% (13/17) of these cases of flu-like illness.

## Discussion

In the 5 years prior to diagnosis, HIV-infected patients had visited their GP more frequently than other healthcare providers, but only one-third of them had one or more HIV tests performed in general practice. More than half of the patients took the initiative themselves for the HIV test that turned out positive.

Almost half of the GPs were not aware of the sexual orientation of newly diagnosed MSM patients, even if MSM themselves thought their GP was aware of their sexual orientation. The sexual orientation was reported in the GP’s EMR in one-third of the MSM group cases. GPs reported that they were aware that the patient was from an HIV endemic country for more than half of the patients in this group.

GPs diagnosed around half of all ICs in the 5 years prior to HIV diagnosis and 40% of these people were offered an HIV test at the time of the IC diagnosis. The majority of patients with a recent HIV infection reported that they had a mononucleosis-like illness, and GPs were frequently contacted about this flu-like illness.

### Comparison with existing literature

Opportunities for provider-initiated HIV testing in primary care prior to HIV diagnosis have been described in previous studies [11, 14–16]. GPs and patients may find it difficult to discuss information about sexual orientation and ethnicity in detail, as this may be perceived as discriminatory or stigmatizing [9, 17–21]. Moreover, there is great diversity in sexual behaviour and identity, even over time for individuals, and MSM may not always identify themselves as such [22]. Our results showed that, despite the belief of the patient, the GP is not always aware of the patient’s sexual orientation. GPs registered the sexual orientation of their MSM patients in the EMR in one-third of the cases. In the Netherlands, no standard information about sexual orientation and ethnicity is recorded in electronic medical records [23]. However, registration of this information may improve the implementation of risk-based testing in primary care. Registration of sexual orientation in the EMR is a sensitive and complex issue [9, 19]. Where this information should be recorded in these files remains a point of discussion. In addition, GPs may not always have knowledge of the country of birth of a patient or connect this with the list of HIV endemic countries [21, 24]. Our results showed that, of the patients who originated in an HIV endemic country, GPs were aware of 56% of the cases. We recommend that opportunities to document this culturally sensitive and gender-specific information in the EMR are created.

European studies have demonstrated that patients with well-established ICs should be recommended to have an HIV test, as these ICs fulfil the criterion of having an HIV prevalence > 0.1% (the threshold for cost-effectiveness) [25, 26]. Another study has confirmed that HIV prevalence is higher than 0.1% for most of the ICs diagnosed in primary care [27]. IC-guided testing is briefly mentioned in the Dutch STI guidelines for GPs [6]. However, a previous Dutch study recommends better integration of relevant ICs in STI guidelines for primary care [20]. Our results found that GPs diagnosed around half of the randomly preselected ICs, and only 40% of this group was offered an HIV test at that time. The strength of IC-guided testing is that it does not depend on HIV risk assessment and could help GPs to bypass some of the barriers presented by complex conversations about sexually risky behaviour or ethnicity [20].

### Strengths and limitations

This study was carried out among diagnosed HIV-infected patients, and despite the number of patients being relatively small, it offers some insight into the health-seeking behaviour of these patients. We combined real-life data from patients and GPs to gain more evidence-based knowledge about how provider-initiated strategies are used by general practitioners.

Dutch or English-speaking HIV-infected patients were eligible for this study. This may have introduced a selection bias, as it is possible that migrants who are at risk of HIV but do not speak these languages may use other health-seeking behaviour.

Finally, information from questionnaires may not reflect all details of the patient’s healthcare history. As data were collected retrospectively, socially desired answers and recall bias cannot be excluded.

## Conclusions

The documentation of sexual orientation, ethnicity and IC-guided testing by GPs could be the starting point for more proactive provider-initiated HIV testing.
